# Vitrectomy and internal limiting membrane peeling for extensive macular schisis in choroideremia

**DOI:** 10.1007/s00417-026-07162-y

**Published:** 2026-03-18

**Authors:** Maram E. A. Abdalla Elsayed, Vincenzo Barone, Robert E. MacLaren

**Affiliations:** 1 Oxford Eye Hospital, Oxford University Hospitals NHS Foundation Trust, Oxford, OX3 9DU UK; 2 Nuffield Laboratory of Ophthalmology, Department of Clinical Neurosciences, University of Oxford, Oxford, OX3 9DU UK; 3 Ophthalmology Complex Operative Unit, University Campus Bio-Medico, Rome, Italy

**Keywords:** Choroideremia, Inherited Retinal Disease, Internal Limiting Membrane Peeling, Macular Schisis, Pars Plana Vitrectomy

## Abstract

**Purpose:**

To report outcomes of pars plana vitrectomy (PPV) with internal limiting membrane (ILM) peeling for extensive macular schisis in choroideremia and provide surgical insights.

**Methods:**

Retrospective case series of five eyes from four patients with choroideremia and extensive macular schisis who underwent PPV with ILM peeling at a single tertiary center between 2011 and 2025. Data collected included pre- and post-operative best-corrected visual acuity (BCVA), surgical technique, spectral-domain optical coherence tomography (SD-OCT) imaging, intraoperative findings, and post-operative complications.

**Results:**

All five eyes showed complete resolution of macular schisis on SD-OCT without foveal retinal pigment epithelium atrophy. BCVA improved in all cases. Intraoperative challenges included retinal fragility, peripheral schisis-like changes, and difficulty with ILM visualization and adherence. No iatrogenic retinal breaks or detachments occurred.

**Conclusion:**

Vitrectomy for extensive macular schisis in choroideremia can yield favourable anatomical and functional outcomes but requires tailored techniques to address retinal fragility and minimize iatrogenic trauma.

## Key Messages

**What is known:**

Extensive macular schisis is a common end stage feature of choroideremia that is frequently missed as patients are no longer being followed up. The schisis however, can detach the small, remaining island of vision leading to potentially treatable vision loss.

Due to retinal fragility, surgical intervention is traditionally considered high risk and of uncertain benefit.

**What is new:**

Pars plana vitrectomy with internal limiting membrane peeling and tailored surgical strategies can achieve favourable anatomical and functional outcomes in choroideremia patients.

## Purpose

 Choroideremia is an X-linked retinal dystrophy caused by mutations in the *CHM* gene, leading to deficiency of Rab escort protein 1 (REP1), which is essential for intracellular trafficking in retinal cells [1–3]. This deficiency disrupts the prenylation of Rab GTPases, resulting in impaired membrane trafficking and progressive degeneration of the retinal pigment epithelium (RPE), choroid, and photoreceptors. Anatomically, degeneration typically begins in the mid-periphery and advances centripetally, with simultaneous loss of RPE and photoreceptors, as shown by high-resolution imaging [4]. As the outer retina and RPE degenerate, the structural support for the overlying retina is lost, leading to collapse and disorganization of the inner retinal layers. This loss of vertical retinal architecture, combined with residual horizontal traction from Müller cells and the inner limiting membrane (ILM), can result in retinal thinning, outer retinal atrophy, and schisis, often culminating in significant visual decline.

 Macular schisis is an uncommon but increasingly recognized manifestation in choroideremia and other inherited retinal diseases (IRD) [5]. It is characterized by multilayered splitting of the macular retina, sometimes extending beyond the vascular arcades, and may resemble the presentation seen in *NR2E3*-associated retinopathies or high myopia.[6–8]. The presence of schisis may worsen central visual function and predispose to further complications, including macular hole (MH) formation and MH-related retinal detachment (RD). Medical treatment options are limited, and surgical intervention remains controversial due to retinal fragility and the typically guarded visual prognosis.

 Since 2012, choroideremia patients have been closely monitored as part of the gene therapy programme, prompting further investigation into the condition’s surgical amenability [9]. In cases of progressive schisis or visual decline, pars plana vitrectomy (PPV) with ILM peeling may provide both anatomical and functional improvement. Here, we present five eyes with macular schisis in choroideremia, managed surgically, and highlight novel intraoperative strategies tailored to the unique challenges of IRD-associated macular pathology.

### Methods

Five consecutive eyes from four male patients (Caucasian) with genetically confirmed choroideremia and spectral-domain optical coherence tomography (SD-OCT) confirmed macular schisis underwent 25-gauge PPV with ILM peeling at a single tertiary care center between 2011 and 2025. Inclusion criteria were progressive vision loss, symptomatic schisis involving the fovea, and no contraindications to surgery; no eligible eyes were excluded. Extensive macular schisis was defined as a multilayer intraretinal schisis involving the fovea with broad horizontal extension affecting at least 25% of the macular cube on SD- OCT.

All patients underwent 25-gauge PPV (core and peripheral vitrectomy), induction of posterior vitreous detachment where necessary, staining of the ILM with Brilliant blue or Membraneblue-Dual^®^, and ILM peeling by a single surgeon. The ILM was peeled in its entirety over the area of the macular schisis, without leaving a flap due to the obvious thickening of the ILM on the pre-operative OCT. The “pick” ILM peel technique was used, employing a 25-gauge cannula (with the tip slightly bent) as a micro-pick to initiate an ILM flap. The 25-gauge cannula was introduced and oriented so that the outer curvature of the tip contacted the retinal surface. Peel initiation was performed away from the foveal centre, in an area of relative structural preservation. The cannula tip was used with very light, tangential strokes to create a small focal “nick” in the ILM, aiming to separate the ILM from the nerve fiber layer without engaging the deeper retina. Once a small ILM edge was elevated, end-gripping forceps were used to grasp the free edge, and a standard continuous peel was performed in a controlled circular fashion, with frequent re-grasping close to the peel margin to minimise traction. If the flap was lost, the bent cannula was re-used to recreate an edge at a nearby site, rather than repeatedly pinching the same fragile area.

20% sulfur hexafluoride (SF_6_) gas tamponade was used. Postoperative care included follow-up at one week, two months, and then annually postoperatively. Data on best corrected visual acuity (BCVA) (LogMAR), SD-OCT images, and complications were recorded. Minimum follow-up was two months.

Written informed consent was obtained from all patients, and all procedures were conducted in accordance with the ethical standards of the Declaration of Helsinki and institutional guidelines.

## Results

**Fig. 1:**
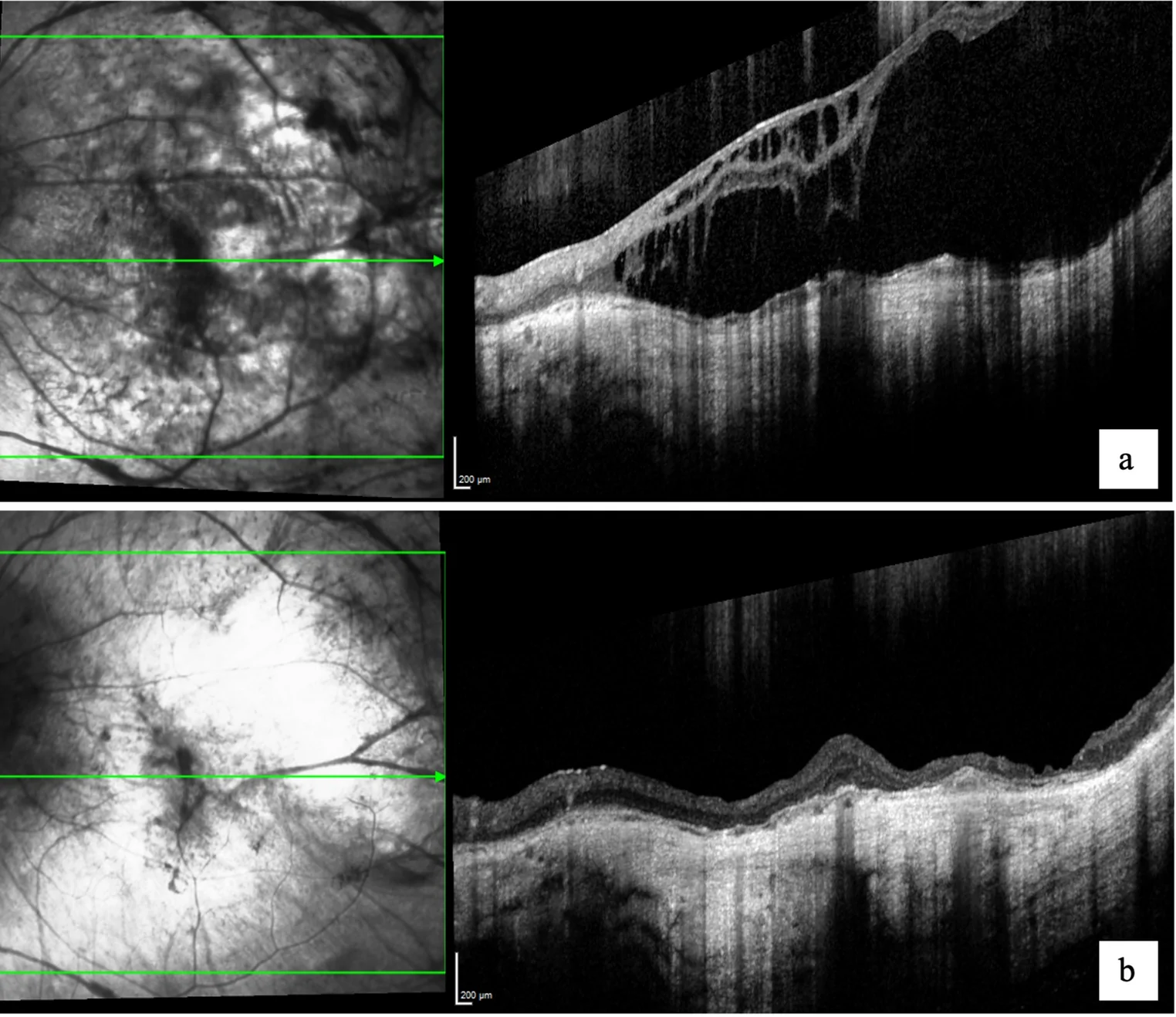
Spectral Domain Optical Coherence Tomography (SD-OCT) scans from Eye 2 (**a**) Preoperative scan showing an extensive macular schisis, primarily involving the outer plexiform layer, with a maximum cavity height exceeding 1074 µm. Visual acuity (VA) was limited to light perception. (**b**) Fourteen years postoperatively, the schisis has completely resolved with restoration of foveal contour. VA improved to LogMAR 1.2

**Fig. 2:**
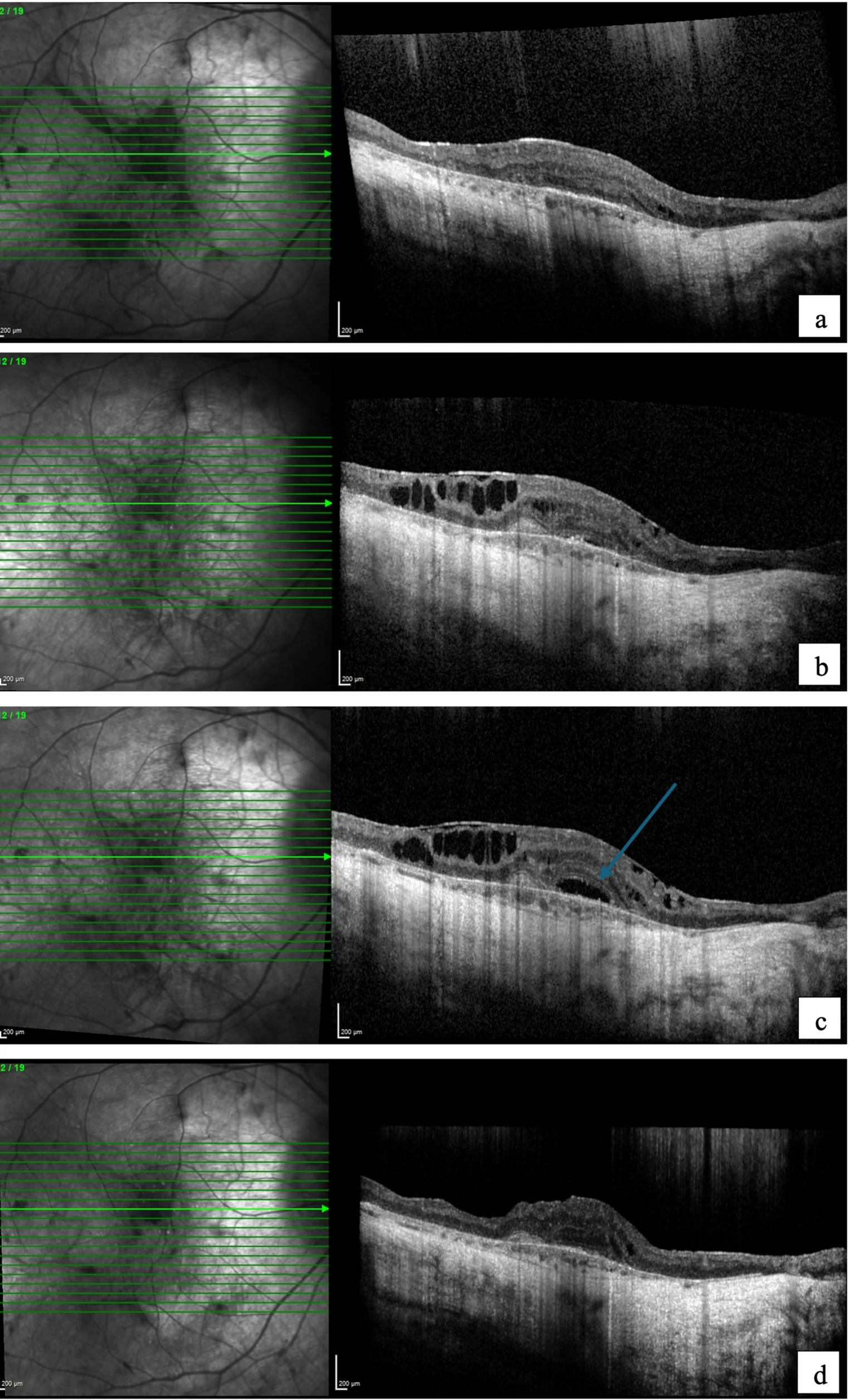
Sequential SD-OCT scans from Eye 5** (a) **Baseline scan prior to the development of macular schisis; visual acuity (VA) was LogMAR 0.6. **(b)** Early stage of schisis formation with initial foveal elevation; VA declined to LogMAR 0.8. **(c)** Progression of the schisis with marked foveal elevation (arrow); VA further declined to LogMAR 1.1. (**d**) Postoperative image at two months showing complete resolution of the schisis and reattachment of the fovea; VA improved to LogMAR 0.8

All five eyes demonstrated complete resolution of the schisis cavities on SD-OCT following surgery with no development of RPE atrophy in the foveal region. Two representative cases are illustrated in Figs. 1 and 2.

**Fig. 3:**
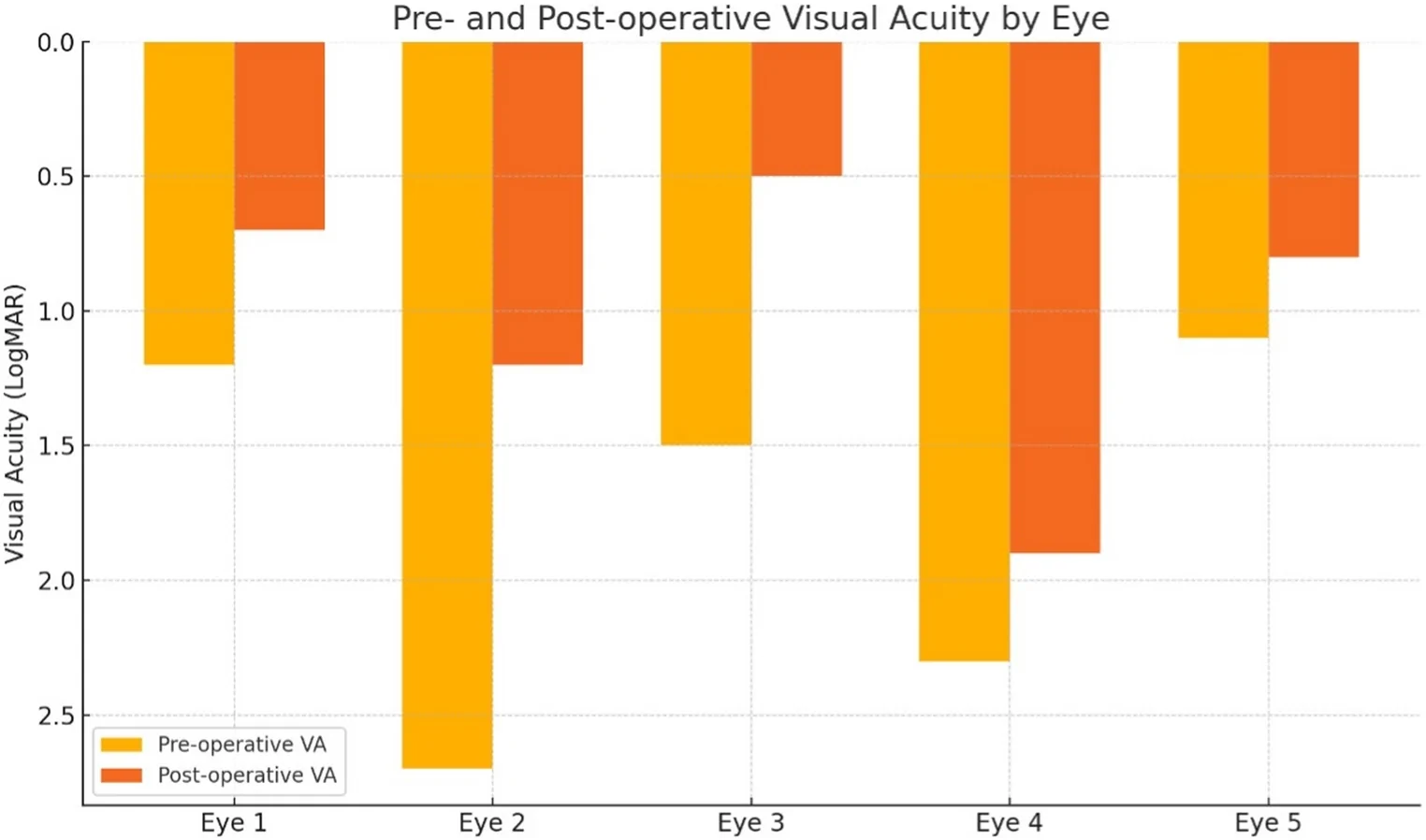
Best-corrected visual acuity for each treated eye before surgery and at two months postoperatively

BCVA improved in all five eyes post-operatively (Fig. 3). Improvement was variable (mean gain 0.74 ± 0.47 LogMAR).

Two eyes (Eye 3 and Eye 5) had previously received subretinal gene therapy - six and four years prior to the onset of macular schisis, respectively; no treatment was administered via the suprachoroidal or intravitreal route, and no ILM peeling was performed at the time of gene therapy. One eye (Eye 4) had presented with a concurrent full-thickness MH pre-operatively and had not undergone prior pars plana vitrectomy before the intervention reported in this study.

Intraoperative challenges reflected the underlying retinal degeneration in choroideremia and included marked retinal fragility, which limited safe manipulation and increased susceptibility to iatrogenic injury. Peripheral schisis-like changes further complicated peripheral vitreous shaving, as intraretinal splitting could mimic shallow retinal detachment and extend with instrumentation. Visualization of the ILM was frequently suboptimal owing to reduced macular contrast and advanced atrophic changes. In addition, the ILM demonstrated firm adherence to the inner retina, making peel initiation technically challenging and increasing the risk of focal inner retinal trauma. No epiretinal membrane formation, retinal tears, retinal detachment, vitreous haemorrhage, or endophthalmitis were observed. Follow-up duration ranged from 2 months to 14 years.

## Discussion

 Macular schisis in choroideremia is rare but increasingly recognized. The pathogenesis likely involves Müller cell dysfunction and vitreomacular traction in the context of progressive retinal atrophy. In our study, there was no difference in the schisis morphology between patients who had received prior gene therapy and those who were treatment-naïve. While visual prognosis in choroideremia is guarded due to outer retinal loss, surgical intervention may be considered in cases of progressive vision loss or schisis-associated complications. Our findings suggest that PPV with ILM peeling can lead to anatomical resolution of schisis and modest functional improvement Choroideremia and related IRD, including *NR2E3*-associated retinopathies, pose unique surgical challenges due to severe retinal thinning, schisis, and diminished elasticity. Degeneration of the RPE and outer retina results in loss of structural support, leading to collapse and disorganization of the inner retinal layers. The absence of vertical retinal architecture and outer limiting membrane, combined with residual horizontal traction from Müller cells and the ILM, contributes to retinal fragility and schisis formation. During peeling, the retina offers minimal resistance. The primary pathology stems from RPE degeneration, which induces secondary photoreceptor and choriocapillaris loss. This cascade culminates in fusion of the degenerate outer retina to the underlying choroid, particularly in the macula and equatorial regions, creating a retinopexy that complicates peeling of the ILM To mitigate the risk of iatrogenic damage, ILM peeling should be limited in radius, ideally confined to the schitic region. In high-risk myopic eyes, ILM peeling under perfluorocarbon liquid is often employed to stabilize the retina and minimize traction, with demonstrated efficacy in preventing foveal rupture during MH and RD surgery [10]. However, this technique is not recommended in choroideremia, where the extremely thin retina may be further compressed by the perfluorocarbon liquid, reducing vertical height and obscuring visualization of the ILM against the pale underlying sclera. Unlike high myopia, which typically preserves photoreceptor structure and maintain RPE reflexes, choroideremia is characterized by advanced photoreceptor and RPE degeneration, further complicating intraoperative assessment. Interestingly, the peripapillary retinal nerve fiber layer (RNFL) in choroideremia remains thickened despite extensive outer retinal degeneration, indicating that *REP1* deficiency does not result in RNFL thinning, in contrast to its effects on outer retinal layers [11]. The preservation of ganglion cells facilitates ILM peeling by providing increased resistance to horizontal traction, thereby enhancing control and precision during membrane dissection Furthermore, visualization of the ILM can be challenging in choroideremia due to underlying RPE atrophy. Brilliant blue or Membraneblue-Dual^®^, applied repeatedly throughout surgery and with prolonged dwell time, is preferred for its favourable toxicity profile and enhanced contrast. Where available, intraoperative OCT can assist in confirming complete ILM removal. As we wish to protect the retina and assist in resolution of the schisis, a long term tamponade would help. Given the notably thin choroid and compromised choroidal perfusion that is characteristic of choroideremia, low concentrations of SF₆ gas are recommended for tamponade. In these patients, 20% SF₆ typically persists intraocularly for an average of eight weeks, obviating the need for longer-acting tamponade agents. Delayed intraocular gas absorption has been reported in these patients, likely due to abnormal retinal architecture and altered intraocular fluid dynamics, which may impede normal gas resorption following surgery [12]. Brilliant Blue or Membraneblue-Dual^®^ facilitates differentiation between neural tissue and Müller cell activation. Choroideremia represents an end-stage retinal degeneration, as demonstrated in the mouse model, and Müller cell activation is evidenced by increased expression of glial fibrillary acidic protein (GFAP). From basic science, it is well established that activated Müller cells contribute to thickening of the ILM, as the ILM represents a continuation of their inner processes. Therefore, in cases where horizontal traction is present, removal of the ILM is entirely logical Our results align with prior studies in high myopia, where ILM peeling has led to schisis resolution [13]. Yu et al. noted that ILM peeling is a risk factor for retinal breaks and MH due to the weakened retinal architecture in the schitic area in X-linked retinoschisis patients [14]. This was not the case in our choroideremia patients. However, visual outcomes in choroideremia are more variable due to baseline photoreceptor loss. Evaluation of the ellipsoid zone and outer retina is critical for preoperative planning and prognosis. Even when vision is not markedly improved, stabilization of structure and prevention of further decompensation may justify intervention 

## Conclusion

 PPV with ILM peeling offers a viable surgical approach for the management of extensive macular schisis in choroideremia, yielding favourable anatomical and functional outcomes. Given the characteristic retinal architecture in choroideremia - marked by thinning, central and peripheral schisis, and pronounced fragility - tailored surgical strategies, including ILM peeling and confining the peel to targeted zones, may enhance intraoperative safety and optimize postoperative results.

## Data Availability

The data supporting the findings of this study are available from the corresponding author upon reasonable request.
